# Deciphering the biosynthesis of a novel lipid in *Mycobacterium tuberculosis* expands the known roles of the nitroreductase superfamily

**DOI:** 10.1016/j.jbc.2023.104924

**Published:** 2023-06-14

**Authors:** Jason C. Grigg, Janine N. Copp, Jessica M.C. Krekhno, Jie Liu, Aygun Ibrahimova, Lindsay D. Eltis

**Affiliations:** 1 Department of Microbiology & Immunology, Life Sciences Institute, The University of British Columbia, Vancouver, British Columbia, Canada; 2 Michael Smith Laboratories, The University of British Columbia, Vancouver, British Columbia, Canada

**Keywords:** actinobacteria, acyltransferase, enzyme, flavin, mycobacteria, *N*-acyl amino acid, NTR, oxazolone

## Abstract

*Mycobacterium tuberculosis*’s (Mtb) success as a pathogen is due in part to its sophisticated lipid metabolic programs, both catabolic and biosynthetic. Several of Mtb lipids have specific roles in pathogenesis, but the identity and roles of many are unknown. Here, we demonstrated that the *tyz* gene cluster in Mtb, previously implicated in resistance to oxidative stress and survival in macrophages, encodes the biosynthesis of acyl-oxazolones. Heterologous expression of *tyzA* (*Rv2336*), *tyzB* (*Rv2338c*) and *tyzC* (*Rv2337c*) resulted in the biosynthesis of C_12:0_-tyrazolone as the predominant compound, and the C_12:0_-tyrazolone was identified in Mtb lipid extracts. TyzA catalyzed the *N*-acylation of l-amino acids, with highest specificity for l-Tyr and l-Phe and lauroyl-CoA (*k*_cat_/*K*_M_ = 5.9 ± 0.8 × 10^3^ M^−1^s^−1^). In cell extracts, TyzC, a flavin-dependent oxidase (FDO) of the nitroreductase (NTR) superfamily, catalyzed the O_2_-dependent desaturation of the *N-*acyl-L-Tyr produced by TyzA, while TyzB, a ThiF homolog, catalyzed its ATP-dependent cyclization. The substrate preference of TyzB and TyzC appear to determine the identity of the acyl-oxazolone. Phylogenetic analyses revealed that the NTR superfamily includes a large number of broadly distributed FDOs, including five in Mtb that likely catalyze the desaturation of lipid species. Finally, TCA1, a molecule with activity against drug-resistant and persistent tuberculosis, failed to inhibit the cyclization activity of TyzB, the proposed secondary target of TCA1. Overall, this study identifies a novel class of Mtb lipids, clarifies the role of a potential drug target, and expands our understanding of the NTR superfamily.

*Mycobacteriumtuberculosis* (Mtb) is the causative agent of tuberculosis (TB), a leading cause of morbidity and mortality worldwide. It was responsible for approximately 1.6 million deaths in 2021, an increase over the 1.2 million deaths in 2019 due to challenges associated with treatment and preventative measures during the COVID-19 pandemic (1). Mtb infects approximately one-third of the world’s population, often persisting as latent infections. Treatment of even drug-sensitive TB requires a cocktail of 3 to 4 antibiotics for approximately 6 months with many toxic side effects (2). This regimen, combined with challenges in accessing prevention and treatment in parts of the world most affected by the disease, resulted in the WHO declaring TB a global health emergency and implementing long-term strategies aimed at eliminating TB (2). Understanding the biology of TB and identifying novel therapies is a key part of this effort.

The extraordinary success of Mtb as a pathogen depends upon its ability to adapt to and modulate its human host, both at the cellular and tissue levels (3, 4). The mechanisms used by Mtb to manipulate the host are not well understood. However, the different host environments place various demands on Mtb, which, in response, senses the environmental shifts and upregulates specific metabolic programs to access nutrients, minimize the consequences of stress, and sustain infection (3, 5). These metabolic programs include the capacity to biosynthesize a rich diversity of lipids, many of which are found in the bacterium’s complex cell envelope of Mtb and contribute to pathogenesis (3). For example, the lipoglycan lipoarabinomannan (LAM) has immunomodulatory effects that contribute to survival and persistence (6). Similarly, glycolipids and glycopeptides (GPLs) contribute to virulence and pathogenesis (6, 7, 8). Despite the important roles of lipids in Mtb biology, the identity, functions, and biosynthetic pathways for many Mtb lipids are not yet determined.

de Rond *et al.*(9) recently characterized a series of acyl-oxazolones produced by *Pseudoalteromonas rubra* DSM 6842 and other γ-proteobacteria. Oxazolones, or Erlenmeyer azlactones, are not commonly described in biology: other than those described in *P. rubra* (9), the only reported examples are the ribosomally produced, copper(I)-binding methanobactins (10), the non-enzymatically-formed-oxazolone in the pentacyclic antibiotic Jadomycin B produced by *Streptomyces venezuelae* ISP5230 (11), and the tryptophan-derived almazolone isolated from red algae (12). In *P. rubra*, OxzA and OxzB specify the biosynthesis of variable-length acyl-tyrazolones (Tyzs) and acyl-phenazolones (9). More specifically, OxzA is an acyltransferase that catalyzes the adenylation of l-Tyr and l-Phe while OxzB is a fusion protein that contains ThiF and nitroreductase (NTR) domains. The ThiF/MoeB/E1 superfamily is comprised of enzymes that catalyze carboxylate adenylation in a wide variety of pathways, including ubiquitination, tRNA maturation, and thiamin biosynthesis (13, 14, 15, 16). The superfamily namesake, MoeB, is a well-characterized enzyme in molybdenum cofactor biosynthesis. A homolog in Mtb, Rv2338c, was predicted to have a similar role and was provisionally annotated as MoeW. Rv2338c was recently identified as the secondary target of the promising anti-TB compound, TCA1 (16). In OxzB, the ThiF and NTR domains catalyze the successive cyclization and desaturation of the acylated amino acids to produce the acyl-oxazolones.

The large NTR superfamily (>34,000 sequences, PFAM fold PF00881) is generally comprised of flavin mononucleotide (FMN)-dependent enzymes that use NAD(P)H to catalyze a wide variety of oxidoreductase reactions (17, 18, 19). Members of the superfamily are typically homodimeric, with the FMN bound in two symmetrical active sites that are formed at the dimer interface. Previously, we used sequence similarity networks and large-scale phylogenetic analyses to delineate the NTR superfamily into 22 major subgroups (17). Taxonomic profiling revealed that four of these subgroups are dominated by sequences originating from Actinobacteria (44–93% of subgroup sequences): the FbiB subgroup that comprises enzymes essential for F420 cofactor biosynthesis (18), and three subgroups of unknown function. One of the latter three, referred to herein as the flavin-dependent oxidase (FDO) subgroup, includes Acg (17). The *acg* gene (Rv2032) is part of the *dos* regulon and is one of the most highly up-regulated genes in the hypoxic model of Mtb dormancy (20). Although the function of Acg is unknown, an Δ*agc* mutant is attenuated in both resting and activated macrophages as well as in acute and persistent murine infection models (21). Crystallographic characterization of Acg from *Mycobacterium smegmatis* revealed strikingly divergent structure and active site architecture: in contrast to the homodimeric enzymes observed in the majority of the superfamily, this family is comprised of monomeric proteins that mimic the homodimeric fold with a single active site (22).

Herein we used sequence similarity networks (SSNs) and phylogenetic analysis to gain better insight into the physiological roles of FDOs in Mtb. Analysis of the genomic contexts of these FDO genes revealed that Mtb FDOs may be involved in the biosynthesis of acyl-oxazolones. We then used the heterologous expression, *in vitro* biochemical assays, and *in vivo* lipid analysis to demonstrate that one of these clusters, *Rv2336*, *Rv2338c-Rv2337c*, hereafter referred to as *tyzA*, *tyzB*, and *tyzC*, respectively, encodes the biosynthesis of acyl-Tyzs. We also tested the ability of TCA1, a small molecule previously identified for its activity against drug-resistant and persistent TB, to inhibit the cyclization activity of TyzB, a proposed secondary target of TCA1 activity (16). The results are discussed in terms of the roles of the biosynthetic cluster in resistance to oxidative stress and survival in macrophages as well as the broader role of FDOs in the physiology and pathogenesis of Mtb.

## Results

### Bioinformatic analysis of the FDO complement of Mtb

**Figure 1:**
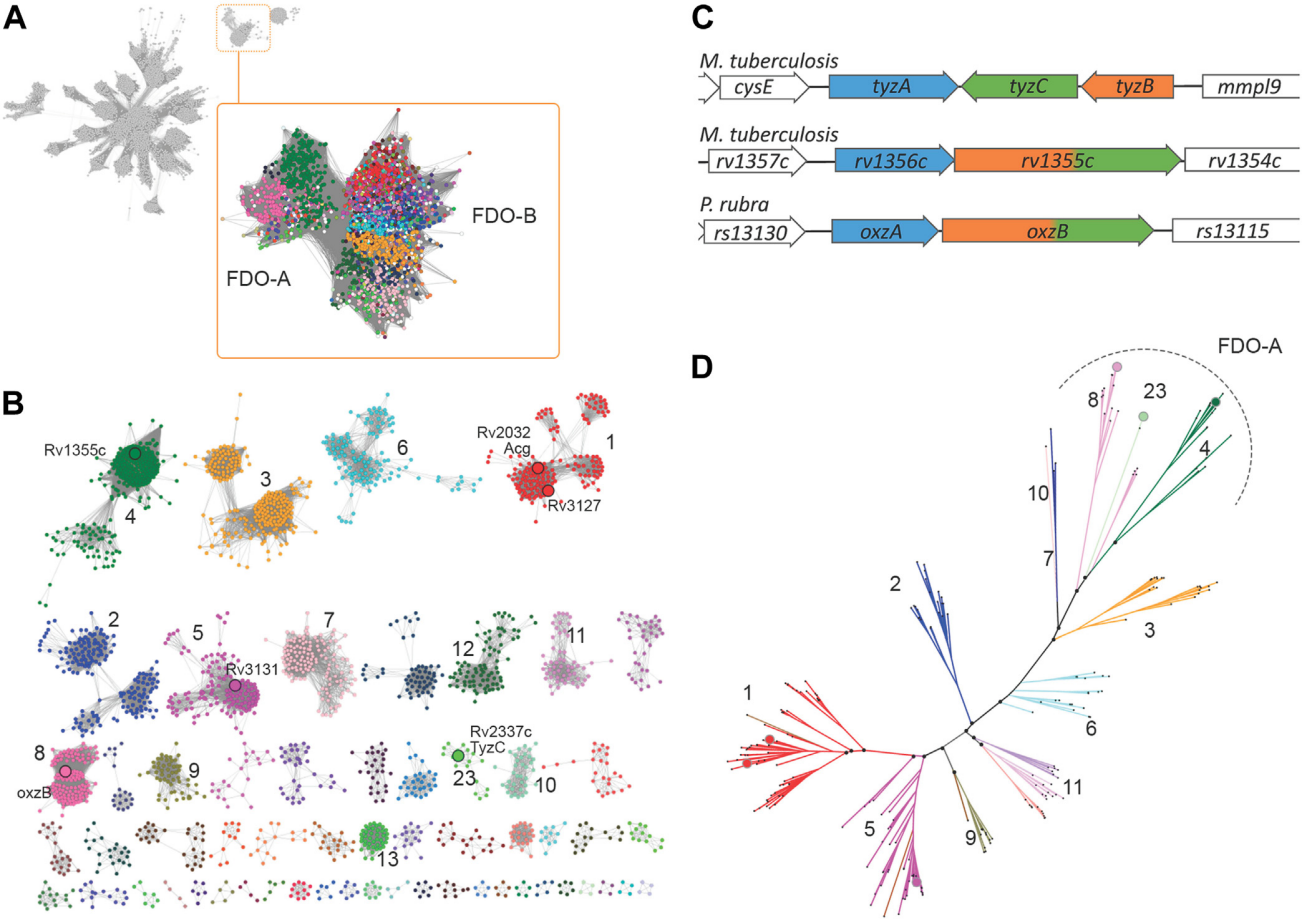
**Bioinformatic analyses of FDOs and the context of Mtb FDO-A genes.***A*, a sequence similarity network (SSN) depicting 5337 nodes that represent proteins that share >60% identity within the nitroreductase superfamily (17). Edges between nodes indicate an average pairwise BLAST *E*-value of at least 1 × 10^−18^. The FDO subgroup is boxed in *orange*. *Inset*: An SSN of 2751 representative nodes representing 6587 proteins of the FDO subgroup that share >50% identity. Edges indicate average BLAST *E*-values of at least 1 × 10^−18^. Node coloring represents subcluster classification. *B*, a SSN of the FDO subgroup delineated into 58 subclusters with >4 nodes at an average pairwise BLAST *E*-value of at least 1 × 10^−72^. The 13 subclusters that encode >200 sequences are numbered. Mtb FDO proteins labeled. *C*, context of the genes encoding FDO-As from Mtb and *P. rubra*. Genes encoding, or predicted to encode acyltransferase (*blue*), FDO-A (*green*) and ThiF (*orange*) domains/proteins are highlighted. *D*, a phylogenetic reconstruction of the FDO subgroup. Branches are colored and labeled by subcluster as indicated in (*B*). Enlarged *black circles* represent branching probabilities >0.85. Enlarged nodes colored by subgroup indicate Mtb FDO sequences, TyzC, Rv1355c, Rv2032, Rv3127, Rv3131, and *P. rubra* OxzA.

The involvement of an FDO in the biosynthesis of acyl-oxazolones in *P. rubra* prompted us to analyze Mtb’s complement of five FDOs. Sequence similarity network analyses demonstrated that the FDO subgroup can be delineated into two highly interconnected sequence clusters, A and B (Fig. 1*A*). At more stringent SSN alignment scores, these two clusters can be delineated into >58 subclusters, 13 of which have >200 sequences (Fig. 1*B*). The two major subclusters of FDO-A, A4 and A8, harbor 14,445 and 623 sequences, respectively (Table S1). FDO-A4 and -A8 also harbor Rv1355c of Mtb and OxzB of *P. rubra*, respectively, both of which are multidomain proteins that include a ThiF protein from the ThiF/MoeB/E1 superfamily (Pfam PF00899). Indeed, the A4 and A8 subclusters contain predominantly longer proteins (>600 aa; Fig. S1) that are predicted to be ThiF-FDO fusion proteins. A second Mtb homolog, TyzC, is found in FDO-A23, a minor subcluster comprising 83 sequences originating from Actinobacteria, Betaproteobacteria, and Hydrogenophilalia. Like the majority of FDOs, which are ∼340 residues in length, TyzC is a single domain protein. However, *tyzC* is adjacent to a gene encoding a ThiF protein, *tyzB*, which is the case for over 50% of genes encoding FDO-A23s for which genomic context is available (Fig. S1). Indeed, the genomic contexts of Rv1355c and TyzC are remarkably similar to that of OxzB (Fig. 1*C*). Thus, not only is Rv1355c predicted to encode a ThiF-FDO fusion protein, but the gene occurs in a putative operon with Rv1356c, a homolog of *oxzA*. Similarly, *tyzC* and *tyzB* occur in a predicted operon that occurs immediately adjacent to, and on the opposite strand as, *tyzA*, encoding an uncharacterized protein. Of the three other Mtb FDOs, Acg (Rv2032) and Rv3127 are found in subcluster FDO-B1, and Rv3131 in FDO-B5 (Fig. 1*B*; Table S1).

### Bioinformatic identification of TyzA, TyzB, and TyzC

Based on their genomic organization and the similarity of the encoded proteins to OxzAB, we hypothesized that the *tyz* gene products specify the biosynthesis of an acyl-oxazolone. Previous studies have implicated these genes in pathogenesis: transposon mutagenesis of *tyzA* attenuated Mtb in macrophage infection (23, 24) and disruption of *tyzC* led to an increased susceptibility to reactive oxygen species (25). In addition, TyzB was identified as a secondary target of the anti-TB compound, TCA1 (16). To test our hypothesis, we first bioinformatically characterized the *tyz* genes and their encoded enzymes in more depth using genomic context analysis, phylogenetic reconstructions, and structural comparisons.

Using the structural model deposited on AlphaFold DB (UniProt: P95233), the 372-residue TyzC was predicted to be a monomeric NTR protein encoding a single flavin binding site with a large lid-like insertion near the active site. In this respect, TyzC appears to be similar to Acg (22) despite these enzymes belonging to FDO subclusters A23 and B1, respectively. To better understand the evolutionary relationships of FDOs, we constructed a maximum-likelihood phylogenetic model using 373 representative sequences. The resulting phylogenetic tree is characterized by highly significant branching probabilities that support the subgroup designations that were identified through sequence similarity networks. More specifically, the tree supports an evolutionary relationship between TyzC and other FDO-As, including the FDO-A4s, and -A8s which are domains of larger fusion proteins (Fig. 1*D*).

TyzA, annotated as a hypothetical 322-residue protein, has 41 homologs, which are predominantly found in mycobacterial strains. Of these 41, 38 have associated sequence information to facilitate genomic context analyses. Of these 38, 25 occur in clusters that are organized similarly to the *tyz* cluster (Fig. S2). A DALI search using the deposited AlphaFold DB model of TyzA (UniProt: P95232) identifies the closest structural homologs as acyltransferases involved in acyl homoserine lactone (AHL) synthesis. These include: FeeM (PDB ID: 2G0B), an acyl-carrier protein-dependent *N-*acyl amino acid synthase identified in a metagenomic clone library; and BjaI (PDB ID: 5W8C), the AHL synthase from *Bradyrhizobium japonicum* that produces isovalerate-homoserine lactone from isovaleryl–CoA and *S*-adenosylmethionine (26, 27). Despite low sequence identity, the AlphaFold model of *P. rubra* OxzA forms the same 3-layer α/β/α structural scaffold. The AlphaFold model of TyzA further indicates that the 30 N-terminal residues may be disordered.

TyzB is a 318 amino acid protein that is part of the large ThiF superfamily (>100,000 sequences PFAM PF00899). Provisionally annotated as MoeW, a molybdopterin biosynthesis protein, the AlphaFold DB model predicts TyzB (UnitProt: P95234) forms an α/β fold with a central 8-stranded β-sheet with peripheral helices. TyzB’s two closest structurally characterized homologs are ThiF and MoeB, both from *Escherichia coli* (13, 14). ThiF and MoeB utilize ATP to catalyze the C-terminal adenylation of their target protein, ThiS and MoeD, respectively.

Analysis of the genomic context of *tyz* genes further revealed that they are flanked by *cysK1* (Rv2334) and *cysE* (Rv2335), which are putatively involved in cysteine biosynthesis, and *mmpl9* (Rv2339). MmpL9 has been implicated in the impairment of phagosome maturation (28). Intriguingly, transposon mutants in *tyzC* and *mmpL9* showed similarly increased susceptibility to oxidative stress (25).

### Heterologous production of TyzA in *Rhodococcus jostii* RHA1

**Figure 2:**
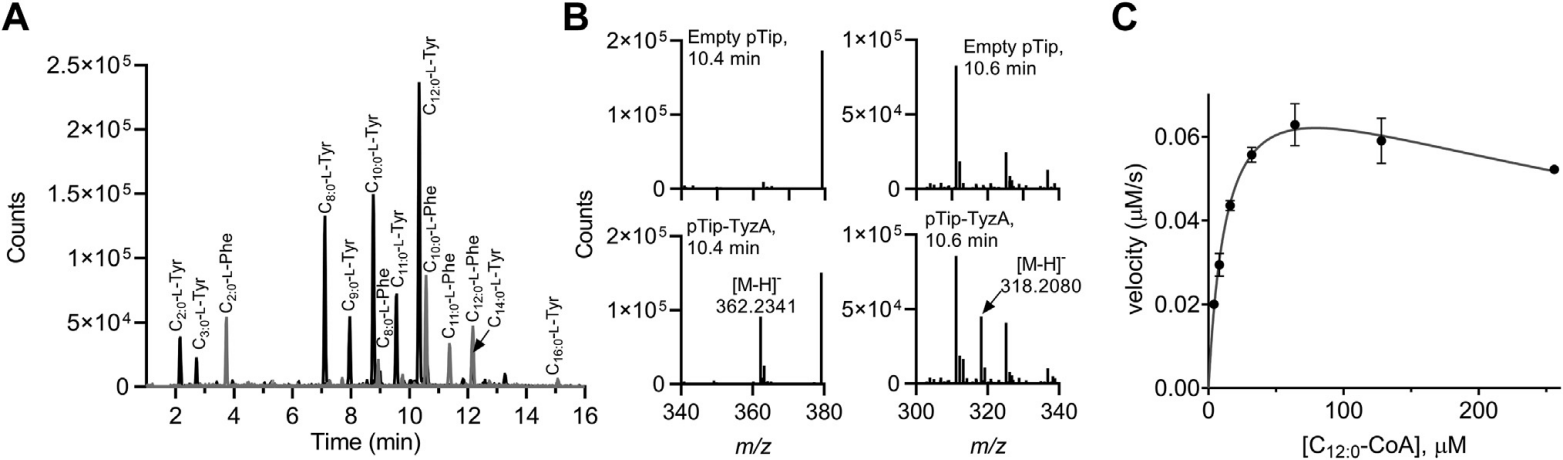
**The activity of TyzA.** TyzA activity was first assessed using RHA1 overproducing the enzyme. *A*, EICs for *m/*z values corresponding to variable length *N-*acyl-L-Tyr (*black traces*) and *N-*acyl-L-Phe (*grey traces*) are shown as detector counts against elution time. For simplicity, only fully saturated acyl species are shown. *B*, mass spectra for WT RHA1 with empty pTip (*top*) or pTip-H_6_-TEV-TyzA (*bottom*) for the peak at ∼10.4 min, corresponding to an [M-H]^-^ = 362.2341 *m/z*, consistent with C_12:0_-L-Tyr and for the peak at 10.6 min corresponding a [M-H]^-^ = 318.2080 *m/z*, consistent with C_12:0_-L-Phe. Values match theoretic masses within 5 ppm error. *C*, steady-state kinetic analysis of purified TyzA with C_12:0_-CoA and l-Tyr. Data points represent the mean and standard deviation of samples in triplicate.

To functionally characterize the Tyz enzymes, we first heterologously produced TyzA in *R. jostii* RHA1 (RHA1). RHA1 is a fast-growing mycolic acid-producing actinobacterium that lacks a homologous TyzABC system. The acyltransferase was expressed using two different systems: a pTip thiostrepton-inducible expression plasmid (29) and an integrative vector, pRIME, under the control of a strong constitutive promoter (30). Metabolites were extracted from cell pellets and supernatants of stationary phase cultures using ethyl acetate. HPLC analysis revealed several features that absorbed strongly at 280 nm that were unique to samples of TyzA-producing cells. Further analysis of the samples using LC-QTOF revealed a striking laddering pattern, particularly in the culture supernatants, with dominant features in the total ion chromatogram (Fig. S3*A*). The most prominent features unique to the TyzA-producing strains were consistent with the expected *m*/*z* (<5 ppm error) of *N*-acyl-L-Tyr and *N*-acyl-L-Phe compounds with acyl chain length varying from C2:0 to C16:0 (Fig. S3*B*). The highest abundance species corresponded to *N*-octanoyl-L-Tyr (C_8:0_-L-Tyr) and *N-*decanoyl-L-Tyr (C_10:0_-L-Tyr). Compounds consistent with the singly desaturated acyl chain were also observed (C10:1 to C14:1), although in lower abundance than their fully saturated counterpart. MS/MS fragmentation was consistent with variable chain length *N*-acyl-L-amino acids, using an *N*-acetyl-L-Phe standard (Fig. S4). Although the laddering pattern was most striking in culture supernatants, extracts from cell pellets suggested the *N*-acyl-L-amino acids were more abundant in the cell than the supernatant and, unlike supernatants, the most abundant form was *N-*lauroyl-L-Tyr (C_12:0_-L-Tyr) (Fig. 2). The wide distribution of chain lengths, together with different degrees of saturation and amino acid identity, is consistent with the observation of multiple species using HPLC. The distribution of species was independent of the expression system used, so all subsequent work was done using pRIME-TyzA.

### TyzA is an acyltransferase

To establish the activity of TyzA, we heterologously produced the enzyme in *E. coli* with an N-terminal H_6_-SUMO-fusion. H_6_-SUMO-TyzA was purified to homogeneity >95%, as judged by SDS-PAGE, and cleaved using SUMO protease (Fig. S5). To assess the activity of TyzA, we first tested its ability to catalyze the *N*-acylation of l-Tyr using lauroyl-CoA (C_12:0_-CoA). Reactions were quenched after 1, 5 and 30 min and analyzed by LC-QTOF. All samples showed the depletion of C_12:0_-CoA and the appearance of C_12:0_-L-Tyr (Fig. S6). Similar rates of reaction were observed for SUMO-tagged and cleaved TyzA, suggesting that the tag does not interfere with the reaction. We further tested the ability of TyzA to use different acyl-CoAs and l-amino acids. The enzyme used all tested CoAs from propionyl-CoA (C_3:0_-CoA) to palmitoyl-CoA (C_16:0_-CoA) and a range of l-amino acids, including l-Tyr, l-Phe, l-Trp, l-Met, and l-Ser. However, TyzA appeared to work most efficiently with C_12:0_-CoA and either l-Tyr or l-Phe (Fig. S7).

To determine the steady-state kinetic parameters of TyzA, we utilized a spectroscopic assay coupling the enzyme reaction to the redox dye, 2,6-dichlorophenolindophenol (DCPIP). The reaction of the blue-colored DCPIP with the free thiol generated from the acyl transfer reaction turns the dye colorless, allowing the progress of the reaction to be monitored by a decrease in absorbance at 600 nm. In this assay, TyzA displayed substrate inhibition kinetics with a *k*_cat_ = 8.3 ± 0.4 × 10^−2^ s^−1^, *K*_m_ = 14 ± 2 μM, *k*_cat_/*K*_m_ = 5.9 ± 0.8 × 10^3^ M^−1^s^−1^, and *K*_*i*S_ = 450 ± 90 μM (Fig. 2*C*). A slight decrease in initial rates at higher concentrations of C_12:0_-CoA was previously observed in BjaI and other AHL synthases where they were presumed to be due to substrate inhibition. The steady-state kinetic parameters of TyzA are on par with those reported for BjaI and FeeM (26, 27).

### Reconstitution of the tyzA-tyzBC gene cluster in RHA1

**Figure 3:**
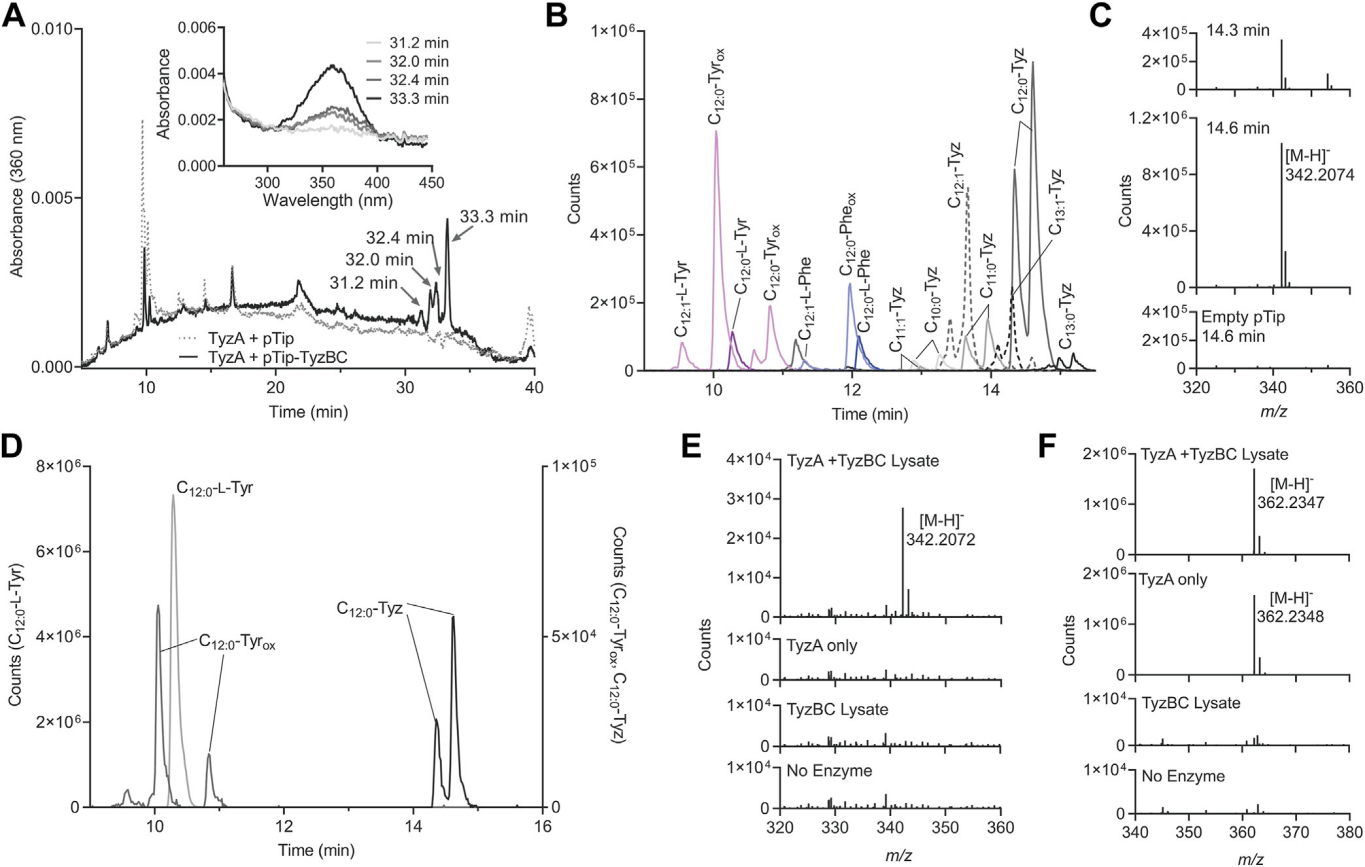
**Reconstitution of the C**_**12:0**_**-Tyz biosynthetic pathway.** The three genes of the pathway were co-expressed in RHA1 and extracts were analyzed using HPLC and LC-QTOF. *A*, HPLC with a phenyl-hexyl column (absorbance at 360 nm shown). Absorption scans of peaks are shown *inset*. *B*, LC-QTOF using a C18 column. Targeted EICs for acylated l-Tyr and l-Phe derived compounds with acylated and oxidized intermediates of l-Tyr and l-Phe shown in shades of *purple* and *blue* respectively. All detected Tyzs are included in varying shades of *grey* or *dashes*, but for simplicity, only C_12:0_-acyl intermediates are shown. *C*, mass spectra for the peaks corresponding to the two isomers of C_12:0_-Tyz (*t*_R_ = 14.3 and 14.6 min) from *panel B*. *D*, EICs for products of the *in vitro* reconstituted pathway using purified TyzA and RHA1 pTip-TyzBC lysates, 0.5 mM l-Tyr, 0.1 mM C_12:0_-CoA, 0.5 mM ATP, and 1 mM MgCl_2_ for approximately 1 h. When TyzBC lysates were omitted from the reaction, no Tyz or desaturated acyl-L-Tyr species were observed. Mass spectra of (*E*) C_12:0_-Tyz (*t*_R_ = 14.6 min) and (*F*) C_12:0_-L-Tyr (*t*_R_ = 10.4 min).

Having established TyzA’s role as an acyltransferase, we sought to reconstitute the entire pathway in RHA1. TyzA was constitutively expressed using the pRIME integrative vector as described above. The *tyzBC* genes were cloned into a pTip vector to maintain their native operonal structure while making use of the pTip promoter. Cells producing TyzA and carrying either empty pTip or pTip-TyzBC were grown to mid-log phase, at which point expression from pTip was induced and cells were grown to the stationary phase (∼24 h) and harvested. Cell pellets were extracted using a butanol-methanol mixture (BUME, (31)) and were analyzed directly using HPLC. Extracts from the pTip-TyzBC carrying strain were yellow in color and yielded several unique HPLC peaks with maxima around 360 nm, consistent with that expected for oxazolone species (Fig. 3*A*). Observation of multiple peaks suggests that at least a few different oxazolone species were produced in this strain.

LC-QTOF also revealed several compounds to be present in the TyzABC-producing strain that were absent in the strain producing only TyzA. These compounds could be classified into two series. The first of these eluted with a slightly lower retention time (*t*_R_) than the corresponding *N*-acyl-L-Tyr/l-Phe product of TyzA with *m/z* values consistent with the predicted TyzC-catalyzed O_2_-dependent desaturation of the substrate (Fig. 3*B*). The TyzC-catalyzed desaturation of the amino acid moiety has the same *m/z* value as the amino acid acylated with a desaturated acyl chain. However, these two classes of compounds have different retention times and can be differentiated based on their MS/MS fragmentation (Figs. S8–S10). The second series of unique compounds had *m/z* values consistent with that of the acyl-oxazolone species. However, only C_9_-C_14_-tyrazolone (Tyz) species were observed, with C_12:0_-Tyz being the most prominent, followed by the desaturated C_12:1_-Tyz (Fig. 3*B*). Despite accumulation of the oxidized acyl-L-Phe, no corresponding phenazolones were observed suggesting that specificity for the modified l-Tyr substrate is dictated by TyzB. MS/MS fragmentation of the C_12:0_-Tyz is consistent with those previously described for acyl-Tyzs (9) (Figs. S9 and S10).

### *In vitro* reconstitution of the pathway

Despite efforts to produce soluble TyzC and TyzB for characterization, both proteins were largely insoluble in *E. coli* and RHA1. However, sufficient amounts of the untagged enzymes were soluble in RHA1 using pTip-TyzBC in WT RHA1, and we were able to assay clarified cell lysates for enzyme activity. Purified TyzA was incubated with lysate from thiostrepton-induced stationary phase RHA1 culture carrying pTip-TyzBC, l-Tyr, C_12:0_-CoA, ATP, and MgCl_2_ for approximately 1 h at room temperature and reaction products were analyzed using LC-QTOF. A compound with the *t*_R_ and *m*/*z* values of C_12:0_-Tyz was only observed when reactions included all of: TyzA, pTip-TyzBC lysates, ATP, and O_2_ (Figs. 3, *D*–*F* and S11). A compound corresponding to the oxidized C_12:0_-L-Tyr (C_12:0:_-Tyr_ox_) was also observed in these samples. These experiments demonstrate that although they were largely insoluble, a small amount of active TyzB and TyzC were present in lysates and performed ATP-dependent cyclization and O_2_-dependent desaturation of C_12:0_-L-Tyr, respectively. Notably, when l-Tyr was replaced by l-Phe or l-Trp in the reaction, neither of their respective Phe or Trp oxazolones were detected.

TyzB, previously annotated as MoeW, had been identified as a possible secondary target of TCA1 (16). Therefore, we next investigated the ability of TCA1 to inhibit TyzB *in vitro*. Reactions were performed as described above but were supplemented with 0, 10 μM, and 100 μM TCA1 prior to initiation. Notably, there was no statistically significant difference in the endpoint abundance for the C_12:0_-Tyz product (Fig. S12) suggesting that, at least in this assay, TCA1 is unable to inhibit the activity of TyzB.

### Identification of C_12:0_-Tyz in Mtb extracts

**Figure 4:**
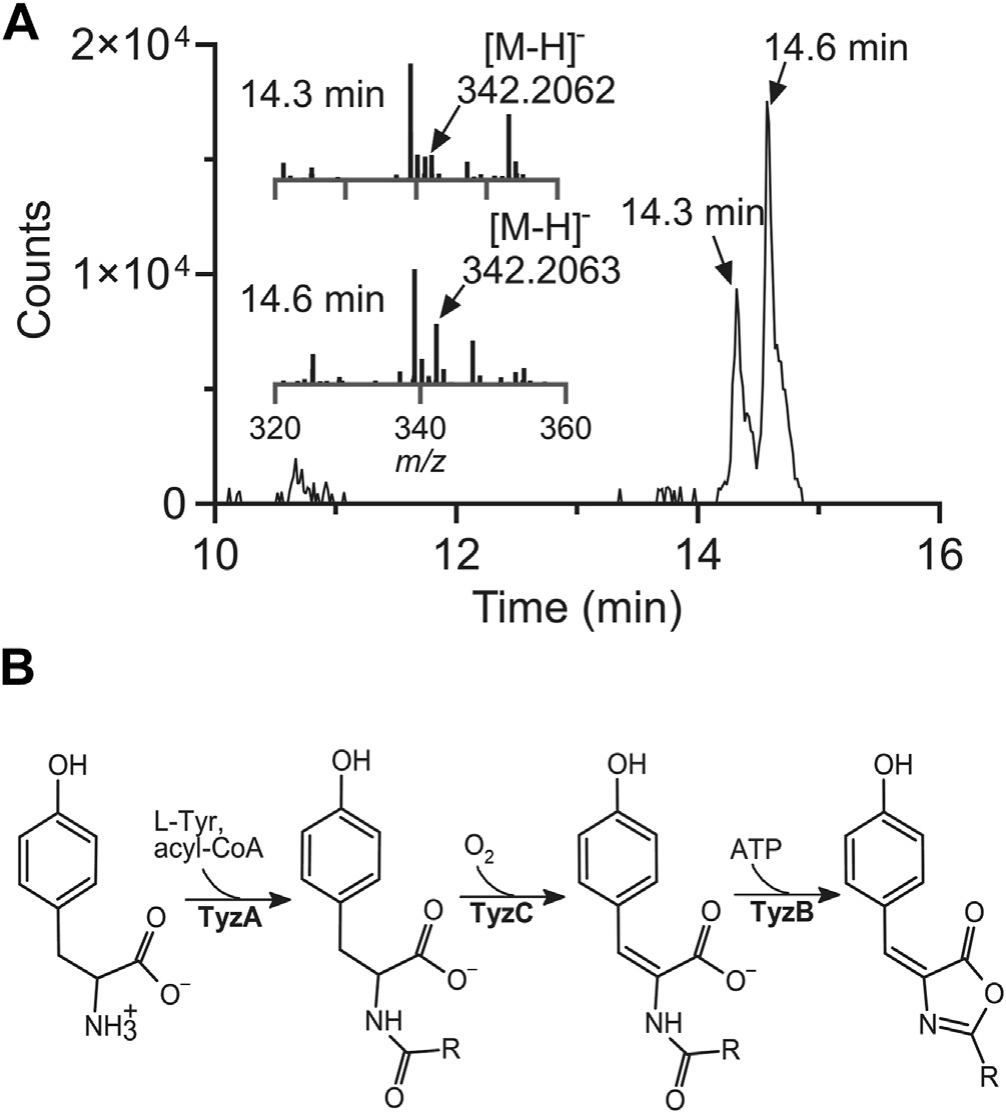
**Acyl-tyrazolone biosynthesis in Mtb.***A*, C_12:0_-Tyz production in Mtb. The EIC for the C_12:0_-Tyz is shown with peak maxima at 14.3 and 14.6 min labeled. Mass spectra for the corresponding peaks are inset with the [M-H]^-^ corresponding to that expected for C_12:0_-Tyz indicated. *B*, the proposed pathway for Tyz synthesis in Mtb.

To determine whether the C_12:0_-Tyz is produced in Mtb, we extracted lipids from *M. tuberculosis* H37Rv Δ*leuCD* Δ*panCD* (mc^2^ 6206) (32). A compound with a *t*_R_ and *m/z* match (<5 ppm error), and the same characteristic double isomer peak for the C_12:0_-Tyz was observed (Fig. 4). MS/MS fragmentation was consistent with that of the C_12:0_-Tyz identified in RHA1 overproducing TyzA, TyzB, and TyzC (Figs. S9*B* and S13). de Rond *et al.*(9) reported that subinhibitory concentrations of antibiotics induce oxazolone production in γ-proteobacteria. Moreover, a transposon mutant in *tyzC* was more susceptible to oxidative stress (25). We therefore tested the effect of isoniazid and NO stress on acyl-Tyz production in Mtb by subjecting cells in 7H9 media to sub-lethal doses of these stresses. Analysis of lipid extracts revealed no obvious differential induction of oxazolone production under either stress condition (Fig. S14). Finally, despite the inability of TCA1 to inhibit the heterologously produced enzyme, we examined whether TCA1 could inhibit TyzB activity *in vivo*. *Mtb* was cultured as described above and with subinhibitory amounts (50% and 20% the MIC) of TCA1. Under these conditions, the amount of C_12:0_-Tyz produced was not significantly lesser (Fig. S15) suggesting that TCA1 does not directly inhibit TyzB.

## Discussion

This study establishes that the *tyz* genes of Mtb encode a pathway responsible for the biosynthesis of an acyl-oxazolone. The pathway comprises an acyl transferase, an FDO-type desaturase, and a ThiF-type cyclase similar to what was recently reported for OxzAB in *P. rubra* (9). Only the C_12:0_-Tyz was detected in Mtb, consistent with this being the predominant species produced by the enzymes *in vitro* as well as by a strain of *Rhodococcus* heterologously expressing the pathway. Nevertheless, these studies indicate that, in Mtb, the pathway likely produces acyl-Tyzs with a variety of chain lengths and some unsaturation. OxzAB produced Tyzs with a similar variety of acyl chains as we observed for the Tyz pathway (*i.e*., C7:0–12:0 and C12:1). Although OxzAB also produced C7:0-C10:0 phenazolones, none were observed from the TyzABC system using either *in vitro* reactions or *in vivo* lipid extractions. Given that TyzA and TyzC appear to acylate and oxidize both l-lyr and l-Phe substrates, and the fact that no cyclized intermediates were observed, it appears that TyzB is the last enzyme of the pathway and determines the specificity for acyl-Tyzs over acyl-phenazolones. Finally, the role of TyzABC in Tyz production agrees with initial annotation of the molybdenum cofactor biosynthetic enzymes in Mtb, which did not include TyzB (MoeW) (33). Given the clear effects of TCA1 on molybdenum cofactor biosynthesis (16), it is unclear whether the inhibitor has additional targets in that pathway or if the systems are somehow linked.

The C_12:0_-Tyz identified in Mtb resembles other key bacterial lipids, namely *N*-acyl amino acids and acyl homoserine lactones (AHL). AHLs, a common signaling molecule in bacteria, comprise a terminal lactone ring with variable acyl-chain lengths typically from C_4_-C_18_ (34). Depending on the acyl-chain length and other substituents, AHLs can either freely diffuse out of the cell (35) or are actively exported (36). The functions of acylated amino acids are less well-defined than AHLs but can act as antibiotics (37), signaling molecules (38), membrane constituents, particularly under phosphate limitation (39), and as components of outer membrane vesicles (40). We have only identified C_12:0_-Tyz in extracts from Mtb cell pellets rather than supernatants. Likewise, when the system is heterologously expressed in RHA1 the products are predominantly isolated from cellular fractions. However, it is still possible that they could be exported or differentially partitioned under specific conditions. The location of the system in proximity to *mmpL9* (Rv2339), encoding a transmembrane transporter of unknown substrate, is intriguing as it could move C_12:0_-Tyz to the bacterial membrane, to vesicles, or for export. However, while a Δ*mmpL9* strain was impaired in its ability to arrest phagosome maturation (28) and resist redox stress (25), transposon-disrupted *mmpL9* strains were not impaired in murine infection models (41, 42). Determining the fate of acyl-Tyzs in the Mtb cell will be important to understanding their function.

This study also greatly expands our understanding of FDOs, a widely distributed family of the NTR superfamily. FDOs are especially well-represented in Actinobacteria, particularly in Mtb, and likely contribute to the pathogen’s adaptability. A notable feature of most Actinobacterial FDOs is their genomic proximity to putative lipid biosynthetic enzymes. Both of the characterized members of the FDO-A subfamily, TyzC of Mtb and OxzB of *P. rubra* (9), catalyze the desaturation of *N-*acyl-amino acids. Based on the homology of these two systems to the third uncharacterized FDO-A containing gene cluster in Mtb, Rv1355c-Rv1356c, and the observation that FDO-As typically co-occur with a ThiF, it is likely that FDO-As broadly catalyzes the desaturation of substrate in the biosynthesis of acyl-oxazolone species. The similarity of Rv1355c to OxzB is intriguing given that both TyzC and OxzB seem to be involved in the biosynthesis of acyl-Tyzs. Determining the product of the second pathway in Mtb will be interesting in evaluating the respective roles of the two oxazolone biosynthetic gene clusters.

The current data indicate that while FDOs-B are not involved in the biosynthesis of oxazolones, they may catalyze the desaturation of other lipid species. More specifically, none of the three genes encoding FDOs-B in Mtb are proximal to other genes predicted to be involved in the biosynthesis of oxazolones. However, all three are part of the DosR regulon: *rv3131* is divergently transcribed from *tgs1*, encoding a triacylglycerol synthase and the most highly up-regulated gene upon the onset of hypoxia (20), and *rv3127* is in the genomic neighborhood. Given the general association of FDOs with lipid biosynthetic enzymes, it seems likely that the FDO family enzymes may catalyze the desaturation of lipid species and may be critical for responding to environmental stresses.

Through characterizing the FDO complement in Mtb, we identified and characterized a Tyz biosynthetic system that appears to be unique to Mtb. The gene cluster had previously been implicated in pathogenesis, suggesting the pathway could be a novel target for TB-specific drug development. Work is ongoing to determine how Tyzs contribute to Mtb physiology and pathogenesis.

## Experimental procedures

### Bioinformatic analyses

NTR superfamily networks were generated as previously described (17). FDO SSNs and genome neighborhood analyses were performed using the EFI Web resource (43) and the PFAM models PF00881 or PF14512 (canonical NTR domains); UniProtKB release 2020_05. Representative sequences for phylogenetic analyses were manually selected as per previously defined criteria (17). Maximum likelihood trees were generated using IQtree (44) with bootstrap approximation and an LG+F + R8 sequence evolution model (45). Structural alignments were generated using the DALI server (46) with structural models from the AlphaFold database (47, 48).

### Bacterial strains and growth media

*E. coli* strains for cloning and *Escherichia cloni* (Lucigen) were grown at 37 °C on Luria-Bertani (5) agar and LB broth supplemented with 30 μg/ml kanamycin. RHA1 was grown at 30 °C and cultured on LB agar and LB broth supplemented with the appropriate antibiotics. Cells containing pTip were supplemented with 30 μg/ml chloramphenicol and those containing pRIME were supplemented with 34 μg/ml apramycin.

### DNA manipulation

Strains and plasmids used in this study are listed in Table S2. Oligonucleotides used in this study are listed in Table S3. FastDigest restriction enzymes and DNA polymerase were purchased from ThermoFisher. The *tyzA* gene (Rv2336) was amplified from Mtb Rv37c genomic DNA using oligos pCDF-Rv2336-F and pCDF-Rv2336-R, and cloned into pCDFDuet-1 at the *Nco*I and *Pac*I sites. The *rv2336* was then excised and ligated into pTip-QC2 at the *Nco*I and *Not*I sites to incorporate an N-terminal His_6_-tag. Subsequently, a TEV-cleavage site was inserted by amplifying from the pTip-H_6_-TyzA template using 2336-TEV-NcF and pTip-II-R primers. The PCR product was digested and ligated at *Nco*I and *Not*I sites to yield pTip-H_6_-TEV-TyzA, which produces TyzA with a TEV-cleavable N-terminal His_6_-tag. Untagged *tyzA* was cloned into the integrative pRIME vector with an M6 promoter (30) using Gibson assembly and the oligos pRIME_Rv2336-F and pRIME_Rv2336-R. For overproduction of TyzA in *E. coli*, *tyzA* was amplified using oligos pExpresso_2336_5p and pExpresso_2336_3p, and cloned into the Expresso SUMO Cloning and Expression System (Lucigen) according to manufacturer recommendations to yield pExpresso-SUMO-TyzA to produce TyzA with an N-terminal H_6_-SUMO fusion. The *tyzBC* genes were amplified from *Mtb* genomic DNA as a single fragment to maintain its operonal arrangement using oligos Rv2338_NdeI-F and Rv2337-XhoI-R, and cloned into pTip-QC2 using Gibson assembly to yield pTip-TyzBC. The nucleotide sequence of all constructs was verified.

### Growth and lipid extractions from RHA1 producing TyzA

To induce expression from pTip, an aliquot from a 4 ml overnight culture in LB with 30 μg/ml chloramphenicol was used to innoculate 25 ml of fresh media to an OD_600_ of ∼0.05 and grown to an OD_600_ of 0.5 to 0.8. Expression was induced by adding thiostrepton to 2 μg/ml, the culture was further incubated for ∼16 h, and the cells were pelleted (4000*g* for 15 min). The supernatant and pellet were extracted using ethyl acetate as previously described (9, 16). Briefly, 5 ml of culture supernatant was decanted into a glass tube, acidified with 0.1% sulfuric acid, and 5 ml of ethyl acetate was added. Extractions were incubated at room temperature for 30 min, and then the lower organic phase was transferred to a clean glass tube and dried under nitrogen gas. The cell pellet was transferred to a glass tube, suspended in 8 ml of ethyl acetate, acidified with 0.1% sulfuric acid, and incubated at room temperature for ∼1 h with vortexing every 15 min to break up cell clumps. Insoluble cell material was removed by centrifugation (1000*g* for 10 min). The soluble fraction was transferred to a clean tube and dried under nitrogen gas. Dried supernatant and pellet extracts were solubilized in 250 μl 35% acetonitrile.

### Recombinant TyzA production and purification

*E. cloni* (Lucigen) with pExpresso-SUMO-TyzA *was* grown overnight in 4 ml LB media supplemented with 30 μg/ml kanamycin. Overnight cultures were diluted 1:500 into 2 × 1 L of LB with 30 μg/ml kanamycin and grown to an OD_600_ of ∼0.8. Gene expression was induced by adding rhamnose to 0.2% and cultures were incubated overnight at 25 °C. Cells were pelleted by centrifugation using an SLK-3000 rotor (4000 rpm for 15 min). Pellets were suspended in 20 ml of 0.1 M Tris, pH 8.0, 0.2 M NaCl, 0.5 mM tris(2-carboxyethyl)phosphine (TCEP), 10 mM imidazole. Cells were lysed using an Emulsiflex C5 homogenizer (Avestin, Ottawa, Canada) and cell debris was pelleted by centrifugation (15,000 rpm for 45 min using an SS-34 rotor). Clarified lysates were loaded on a 5 ml Ni^2+^-NTA column (Qiagen), washed with five column volumes of suspension buffer supplemented with 50 mM imidazole, and eluted with the same buffer supplemented with 300 mM imidazole. Elution fractions were analyzed by SDS-PAGE and dialyzed into 20 mM Tris, pH 8.0, 0.2 M NaCl, 0.5 mM tris(2-carboxyethyl)phosphine (TCEP). Purified H_6_-SUMO-TyzA was concentrated to 10 mg/ml using an Amicon 10 MWCO spin column and flash frozen in liquid nitrogen as beads. Protein was cleaved by overnight digestion at 4 °C with SUMO protease (Lucigen). TyzA was separated by passing cleavage products over a 5 ml Ni^2+^-NTA column and collecting the flowthrough. Cleaved TyzA was concentrated, and flash frozen as described above for future use.

### Production of TyzC and TyzB in RHA1

pTip-TyzBC was transformed into WT RHA1 and protein was produced as described above for TyzA in RHA1.

### *In vitro* endpoint reactions

Endpoint TyzA reactions were set up using SUMO-tagged and cleaved protein from concentrated stock. The 200 μl reaction mixture consisted of 20 mM MOPS, pH 7.2, 80 mM NaCl, 0.5 mM l-amino acid, 0.2 mM acyl-CoA and 1 μM TyzA. Reactions were initiated by adding enzyme and quenched by the addition of LCMS-grade acetic acid to 10%. Samples were then centrifuged in a microfuge for 10 min at 16,000*g*, transferred to glass HPLC vials and analyzed using LC-QTOF, as described below. Endpoint reactions for the entire TyzABC system were set up as above, with 20 mM MOPS, pH 7.2, 80 mM NaCl, 0.5 mM l-Tyr, 0.2 mM C_12:0_-CoA, 0.5 mM ATP, 1 mM MgCl_2_ and were initiated with the subsequent addition of 1 μM TyzA and then 15 μl of clarified RHA1 pTip-TyzBC lysate in a final volume of 200 μl. For TCA1 inhibition studies, TCA1 was dissolved in DMSO and added to the reaction mixture (0, 10 μM or 100 μM) prior to initiation of the reaction in triplicate.

### Steady-state kinetics for TyzA

Steady-state kinetic parameters were determined using 2,6-dichlorophenolindophenol (DCPIP) to detect the free thiol liberated during acyl-transfer. Reaction mixtures contained 20 mM MOPS, pH 7.2, 80 mM NaCl, 1.0 mM l-Tyr, 5 to 250 μM C_12:0_-CoA, 150 μM DCPIP. Reaction mixtures were prepared without enzyme and incubated for 5 min to reduce background reaction of DCPIP. Reactions were initiated by the addition 1 μM H_6_-SUMO-TyzA. Reactions were monitored using a Cary 5000 spectrophotometer equipped with a thermostatted cuvette holder at 25 °C. The reaction was monitored at 600 nm using an extinction coefficient of 21,000 M^−1^ cm^−1^. We verified that DCPIP was not rate-limiting by performing the experiment at different DCPIP concentrations. Steady-state kinetic parameters were determined by fitting a model for substrate inhibition (49) (Equation 1) using GraphPad Prism.

(1) $$V=\frac{V_{\max}\left[S\right]}{K_{m}+\left[S\right]\left(1+\frac{\left[S\right]}{K_{i}}\right)}$$

### Growth and lipid extractions from RHA1 containing TyzA, TyzB, and TyzC

RHA1 strains containing pRIME-M6-TyzA (where M6 is a constitutive promoter) and pTip-TyzBC were grown on LB supplemented with 30 μg/ml chloramphenicol and 34 μg/ml apramycin. Expression of *tyzBC* was induced as described above for pTip. Lipids were extracted using a modified BUME method (31). Briefly, cell pellets from ∼10 ml of culture were transferred to glass tubes and suspended in 0.5 ml of a 1:1 (v/v) mixture of 1-butanol:methanol supplemented with 5 mM ammonium formate, pH 6.5 (2.5 μl of a 1 M stock), vortexed to suspend cells, and sonicated in a sonicating water bath for 30 min, vortexing every 10 min to break up cell clumps. Insoluble debris was then pelleted (1000*g* for 10 min) and transferred to a clean glass tube. Samples were analyzed directly by HPLC or LC-QTOF.

### HPLC analysis of extracts

HPLC analysis was performed on a Waters 2695 HPLC equipped with a Waters 2996 photodiode array detector (Waters). 50 μl extract was injected onto a 250 × 4.6 mm Luna 5 μm phenyl-hexyl column (Phenomenex) and separated on a 35 min linear gradient from 5 to 100% acetonitrile with 0.1% formic acid at a flow rate of 1 ml/min. Data were collected and analyzed using Waters software and plotted using Graphpad Prism 9 (GraphPad Software).

### LC-QTOF

LC-MS analysis was performed using an Agilent 1290 Infinity II UHPLC in line with an Agilent 6546 Q-TOF with a dual AJS ESI source. Two μl samples were injected onto a Zorbax Eclipse Plus C18 column (100 mm × 2.1 mm × 1.8 μM) and run on a 16-min linear gradient from 5 to 100% solvent B at 0.45 ml/min. Solvent A was 0.1% formic acid in water, Solvent B was 0.1% formic acid in acetonitrile. MS parameters in negative ionization mode were as follows: capillary voltage, 4000 V; nozzle voltage, 2000 V; drying gas temp, 300 °C; drying gas flow rate, 10 L/min; sheath gas temperature, 350 °C; sheath gas flow rate 12 L/min, nebulizer pressure, 45 psi; fragmentor voltage, 100 V. Parameters for positive ionization mode were the same, except capillary voltage, 3500 V and nozzle voltage, 500 V. MS/MS was collected on selected ions with 10, 20, and 40 V collision energies. Data were collected and analyzed using MassHunter Workstation Version 10 (Agilent Technologies).

### Oxazolone accumulation in *M. tuberculosis*

Mtb H37Rv mc^2^ 6206 was grown in 100 ml 7H9 containing 0.05% Tween 80, 0.2% glycerol, 10% OADC, 24 μg/ml pantothenate, and 50 μg/ml leucine in standing tissue culture flasks at 37 °C. Duplicate volumes of 10 ml were pelleted *via* centrifugation (4000*g* for 10 min) and suspended in 10 ml 7H9 containing 0.05% Tween 80, 0.2% glycerol, 10% OADC, 24 μg/ml pantothenate, and 50 μg/ml leucine with or without a stressor: 1.5 mM NaNO_2_, pH 5.5 or 0.05 μg/ml isoniazid (INH). These new cultures were started at an OD_600_ of 1.3 and incubated in standing tissue culture flasks for 4 days at 37 °C. Before harvesting *via* centrifugation (4000*g* for 10 min), OD_600_ was measured. Culture pellets were stored at −80 °C until lipid extraction. Cellular lipids were extracted by suspending 10 ml-culture pellets in 400 μl of 1-butanol-methanol (1:1) supplemented with 5 mM ammonium formate, pH 6.5 (2.5 μl of a 1 M stock) and vortexing. Samples were then sonicated for 40 min with vortexing every 10 min. Extracts were then isolated *via* centrifugation and removed from pelleted debris. Samples were dried under N_2_ gas, solubilized in 100 μl of 1-butanol-methanol (1:1) and analyzed using LC-QTOF, as described above.

For experiments with TCA-1, Mtb H37Rv mc^2^ 6206 was grown in 30 ml 7H9 containing 0.05% Tween 80, 0.2% glycerol, 10% OADC, 24 μg/ml pantothenate, and 50 μg/ml leucine in standing tissue culture flasks at 37 °C. Using this culture, duplicate volumes of 50 ml of 7H9 containing 0.05% Tween 80, 0.2% glycerol, 10% OADC, 24 μg/ml pantothenate, and 50 μg/ml leucine with 0, 38, or 95 μg/ml TCA-1 were inoculated to an OD_600_ of 0.15. The cultures were incubated in roller bottle flasks at 40 rpm for 3 days at 37 °C until an OD_600_ of ∼1 was reached. Bacteria were harvested *via* centrifugation (4000*g* for 10 min) and pellets were stored at −80 °C until lipid extraction. Cellular lipids were extracted by suspending each pellet in 500 to 678 μl (volume normalized by OD) of 1-butanol-methanol (1:1) supplemented with 5 mM ammonium formate, pH 6.5 (2.5 μl of a 1 M stock) and treated as described above prior to analysis by LC-QTOF.

## Data availability

All data are contained within the manuscript and supporting information.

## Supporting information

This article contains supporting information (9, 16, 29, 30, 32, 43, 50).

## Conflict of interest

The authors declare that they have no conflicts of interest with the contents of this article.
