# Supplementary material for: Deciphering the biosynthesis of a novel lipid in Mycobacterium tuberculosis expands the known roles of the nitroreductase superfamily
Source: J Biol Chem. 2023 Jun 14;299(7):104924. doi: 10.1016/j.jbc.2023.104924 (PMC10404671; doi:10.1016/j.jbc.2023.104924)
Supplement: Supporting Figures S1–S15 and Tables S1–S3 [file mmc1.docx]

**Supplementary Information**

**
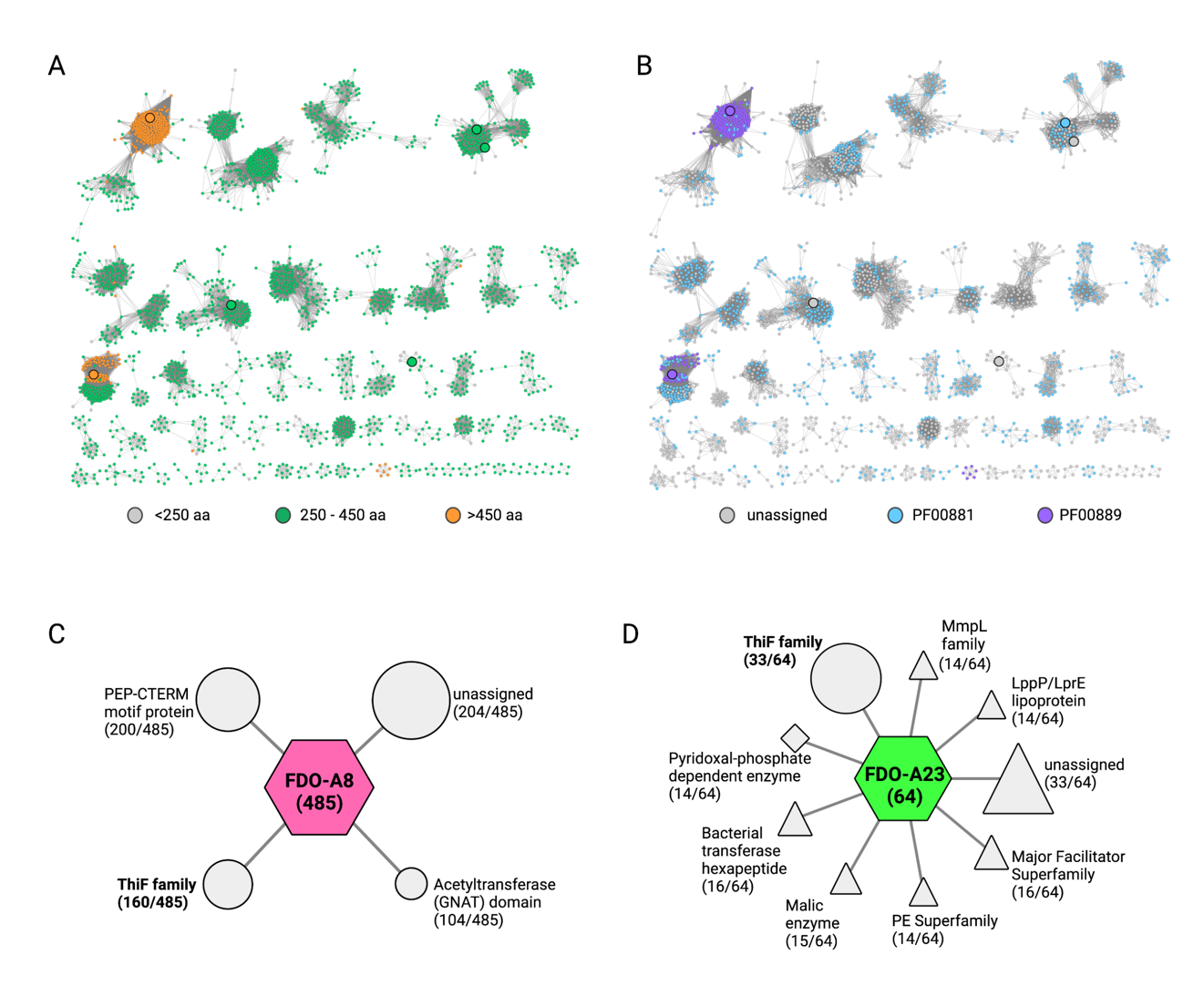
**

**Figure S1. Sequence similarity networks (SSN) and genomic neighborhood analysis (GND) of FDOs**. (A) A SSN of the FDO subgroup delineated into subclusters at an average pairwise BLAST *E*-value of at least 1×10^-72^. Nodes are colored by average sequence length. Mtb FDO proteins are indicated by enlarged nodes (labeled in Fig. 1B). Clusters containing <4 nodes were omitted for clarity. (B) SSN as per A, colored by PFAM designation: PF00881 (NTR superfamily) and PF00889 (ThiF superfamily). Clusters containing <4 nodes were omitted for clarity. (C and D) GND of FDO-A8 (c) and FDO-A23 (d), depicted as hub (SSN subcluster) and spoke (PFAM family) diagrams, depicting the co-occurrence frequencies of queries and their genome neighbors. The number of neighbors with available genomic context is indicated in parentheses in the hub nodes; the co-occurrence ratio is indicated in parentheses in the spoke nodes.

**
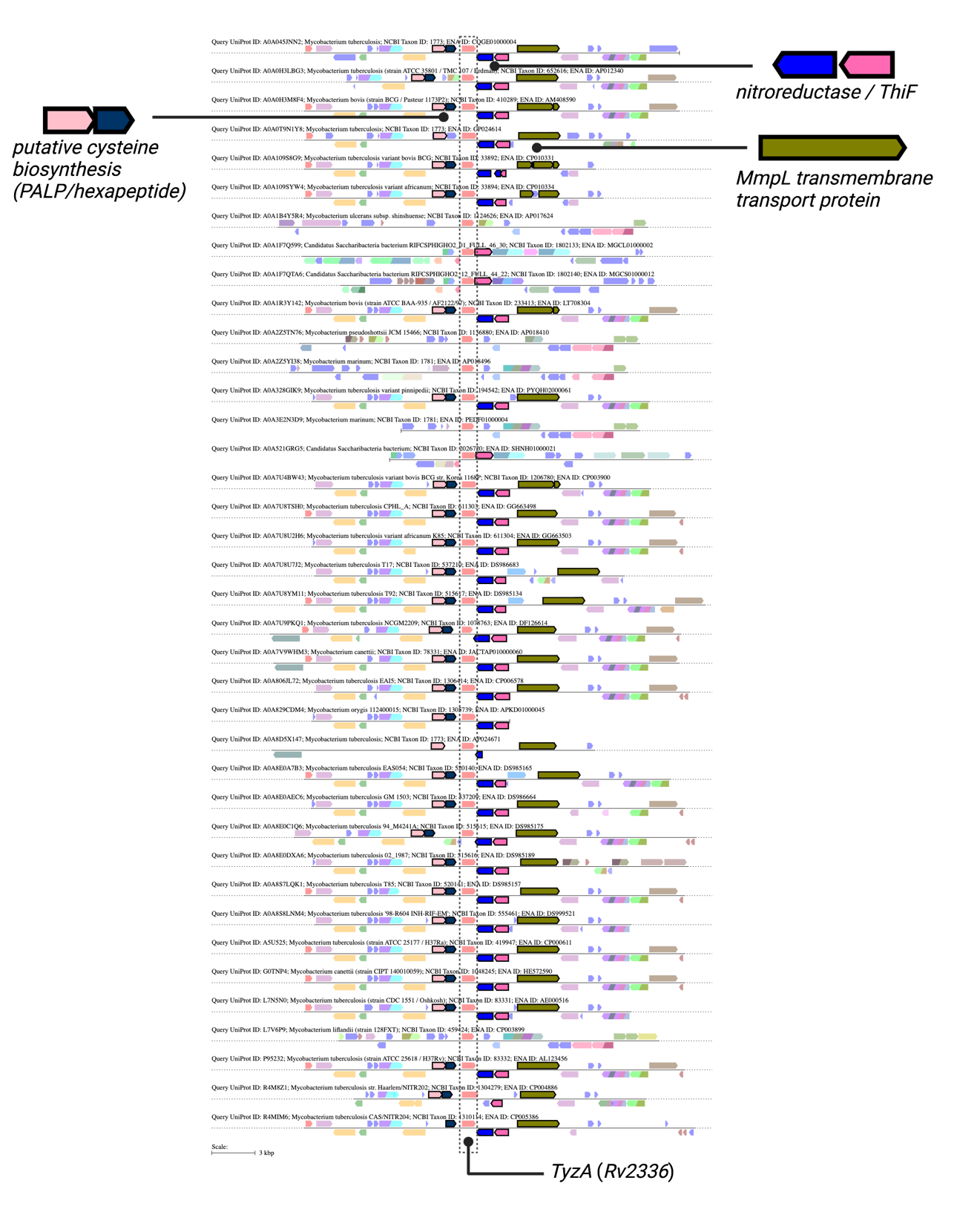
Figure S2. A genomic neighborhood diagram (GND) of TyzA (Rv2336) homologs**. TyzA homologs colocalized with genes annotated as nitroreductases, ThiF enzymes, transmembrane transport proteins and putative cysteine biosynthesis enzymes. The data were visualized using GND Explorer (43).

**
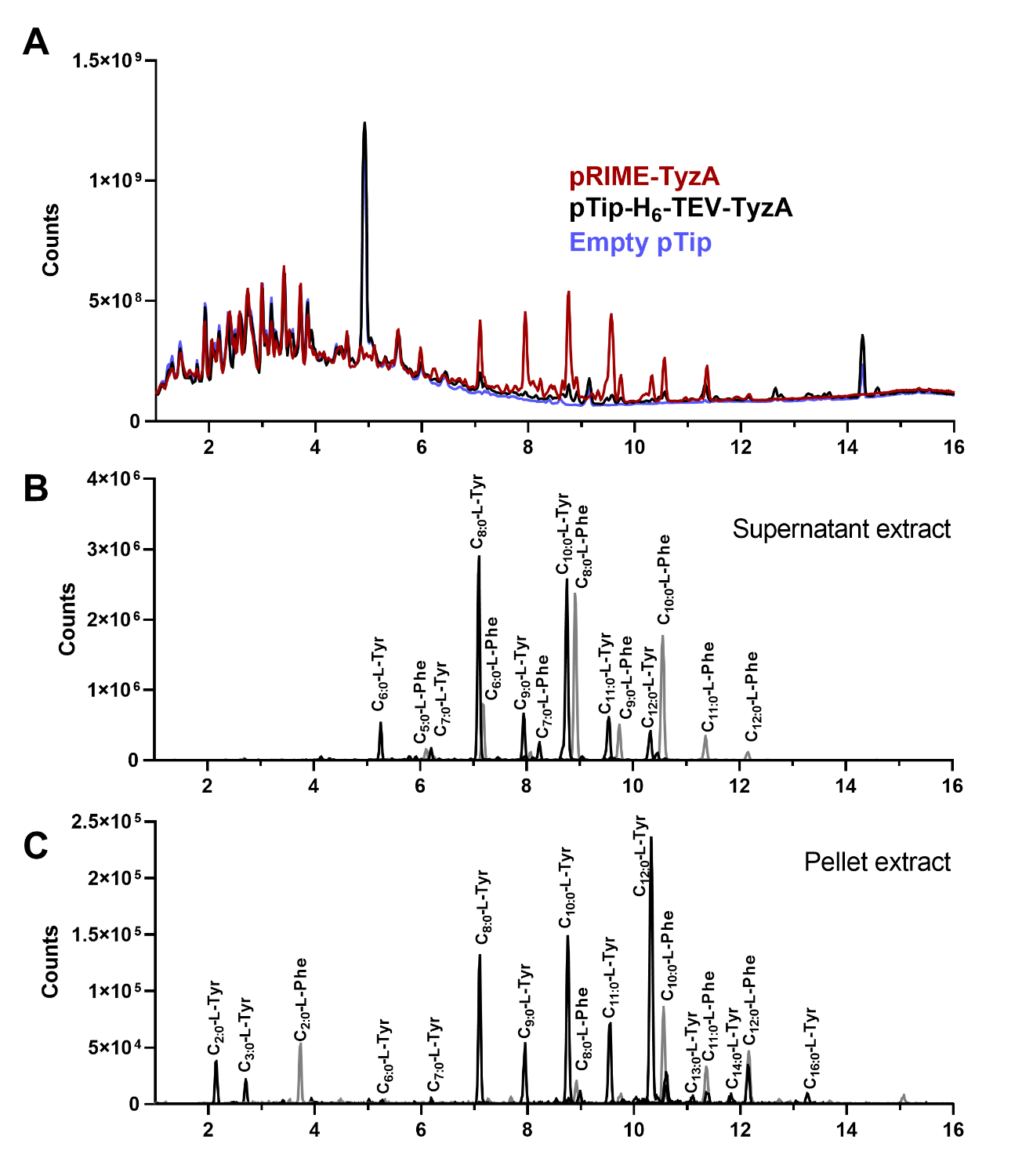
**

**Figure S3. LC-QTOF comparison of acylated L-Tyr and L-Phe produced in RHA1 strains heterologously producing TyzA**. (A) Total ion chromatograms for culture supernatants of RHA1 strains producing TyzA from pTip-H_6_-TEV-TyzA (black), pRIME-TyzA (red), or an empty pTip control. The large peak at ~5 min in the pTip and pTip-H6-TEV-TyzA is due to the thiostrepton added to cultures and is independent of TyzA expression. Extracted ion chromatograms for the *m*/*z* values corresponding to different chain-length acyl-L-Tyr and acyl-L-Phe species from (B) culture supernatants and (C) cell pellets of RHA1 with pTip-H_6_-TEV-TyzA.


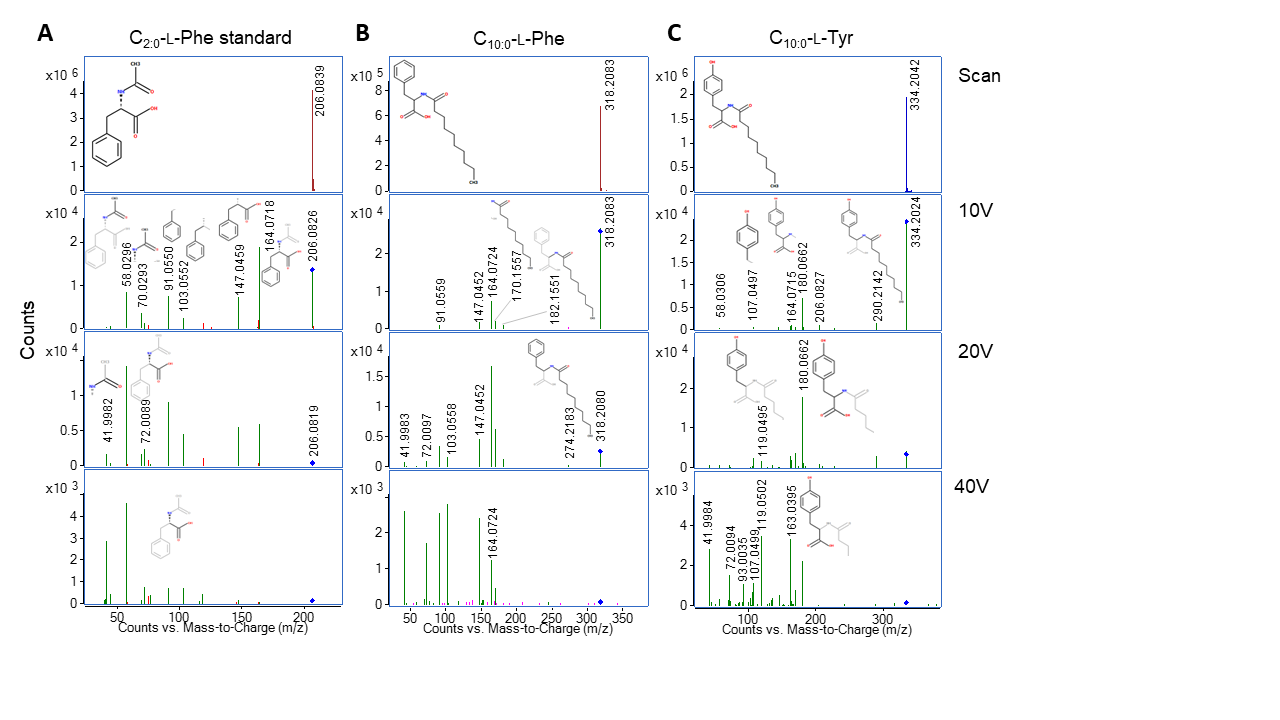
**Figure S4. MS/MS fragmentation of the TyzA reaction products from culture supernatants.** MS/MS was performed on (A) an authentic C_2:0_-L-Phe standard and compounds with *m*/*z* values matching (B) C_10:0_-L-Phe and (C) C_10:0_-L-Tyr. Compounds were extracted from culture supernatants of *R. jostii* RHA1 producing TyzA.

**Figure S5. SDS-PAGE analysis of TyzA preparation**. Samples are: Uncut, H_6_-SUMO-TyzA recovered from initial Ni^2+^-Sepharose column; Load, sample after incubation with SUMO protease; Flow-through, cleaved sample after passage over Ni^2+^-Sepharose column; and Elute, the 0.3 M imidazole wash of resin to remove any bound material from the sample after SUMO protease treatment. The presence of cleaved TyzA suggests the protein has some affinity for the resin. Only the flow-through fraction was concentrated and used in downstream analysis.

**Figure S6. Activity of TyzA.** (A) LC-QTOF analysis of reactions containing 0.5 µM TyzA, 0.2 mM C_12:0_-CoA and 0.5 mM L-Tyr. Reactions were quenched at 1, 5 and 30 min after enzyme addition and analyzed by LC-QTOF. EICs for the *m/z* value corresponding to C_12:0_-L-Tyr are shown. Mass spectra for the peak areas are inset.

**Figure S7. Acyl transfer activity of** H_6_-**SUMO-TyzA in *E. coli* lysates.** LC-QTOF analysis of TyzA reactions performed as described in the Methods, with the following reaction components: 10 µL clarified cell lysate from *E. coli* containing H_6_-SUMO-TyzA; 0.2 mM of the indicated acyl-CoA; and 0.5 mM of the indicated L-amino acid. Reactions were incubated at 25°C for 2 hours and analyzed using LC-QTOF. The EICs correspond to the *m*/*z* values for the respective *N*-acyl-L-amino acid. Mass spectra for the peak areas inset.

**Figure S8. Differentiation of TyzA and TyzC reaction products possessing the same *m*/*z* values.** The *m*/*z* value of C_12:1_-L-Tyr (a reaction product of TyzA) is the same as that of the TyzC-catalyzed oxidation product of C_12:0_-L-Tyr. The two compounds were differentiated using MS/MS fragmentation. Briefly, the peak with an *m*/*z* value at 180.067 represents loss of the *N-*acyl chain and is observed in the compounds with *t*_R_ of 9.6 min and 10.3 min for C_12:0_-L-Tyr. The fragment at 316/318 represents a loss of CO_2_. This allows assignment of the 9.6 min peak as C_12:1_-L-Tyr. The compounds with *t*_R_ of 10.0 and 10.8 min possess similar fragmentation patterns and are consistent with the two isomers of the TyzC oxidation product of C_12:0_-L-Tyr. The compound with a *t*_R_ of 10.6 min was not readily assigned.

**A**

**B**

**Figure S9. LC-QTOF analysis of extracts from *R. jostii* RHA1 containing TyzA, TyzB and TyzC.** (A) EICs for saturated acyl-L-Tyr and acyl-L-Phe reaction products. (B) Negative mode MS/MS Fragmentation of the compounds corresponding to C_12_-L-Tyr (10.3 min), oxidized C_12_-L-Tyr (10.0 min) and the two C_12:0_-Tyz isomers (14.3 and 14.6 min). Predicted fragments are shown as inset structures next to their corresponding *m*/*z* label, with the fragment bonds in black. Structures have been cropped, where required, to fit within the window. Likely fragment structures were determined using Agilent Molecular Structure Correlator software. Where multiple structures were possible, the highest scored option is shown. The Y-axis indicates detector counts and an MS scan and 10, 20 and 40 V fragmentation energies are shown for each compound.

**Figure S10. Positive Mode MS/MS fragmentation of C_12:0_-L-Tyr metabolites produced using *R. jostii* RHA1 containing TyzA, TyzB and TyzC.** Columns correspond to C_12:0_-L-Tyr (10.3 min), C_12:0_-Tyr_ox_ (10.0 min) and the two C_12:0_-Tyz isomers (14.3 and 14.6 min) from Fig. S7. The Y-axis indicates detector counts and an MS scan and 10, 20 and 40 V fragmentation energies are shown for each compound with *m*/*z* values labeled. Fragments for the C_12:0_-Tyz species are consistent with those described by de Rond *et al.* (9).


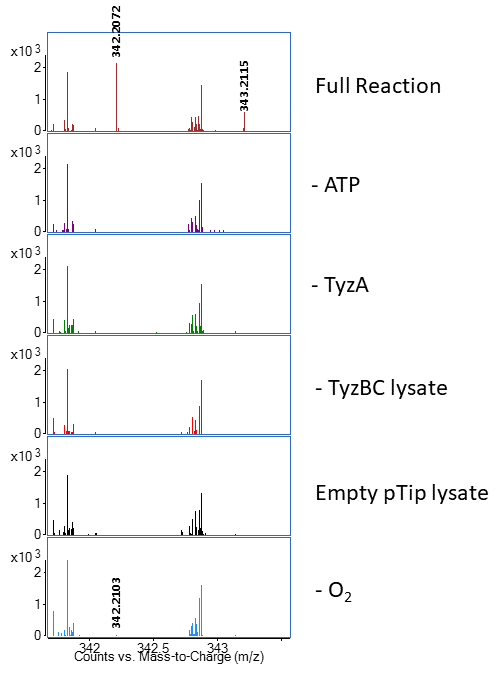


**Figure S11. ATP and O_2_-dependence of C_12:0_-Tyz production by TyzABC.** Negative mode mass spectra corresponding to the expected *m/z* (342.2075) for the C_12:0_-Tyz. A compound corresponding to the C_12:0_-Tyz (<1 ppm error) was only clearly observed for the full reaction containing 20 mM MOPS, pH 7.2, 80 mM NaCl, 0.5 mM L-Tyr, 0.2 mM C_12:0_-CoA, 0.5 mM ATP, 1 mM MgCl_2_. When ATP (-ATP), TyzA (-TyzA), TyzBC (-TyzBC and Empty pTip lysate) were omitted, no compounds corresponding to C_12:0_-Tyz were observed at the expected retention time. When the reaction was performed in a glove box with nitrogen atmosphere, a small peak ~1% of the full reaction peak height consistent with the expected *m/z* (<10 ppm error) was observed and may correspond to reaction with trace O_2_ present in enzyme solutions.


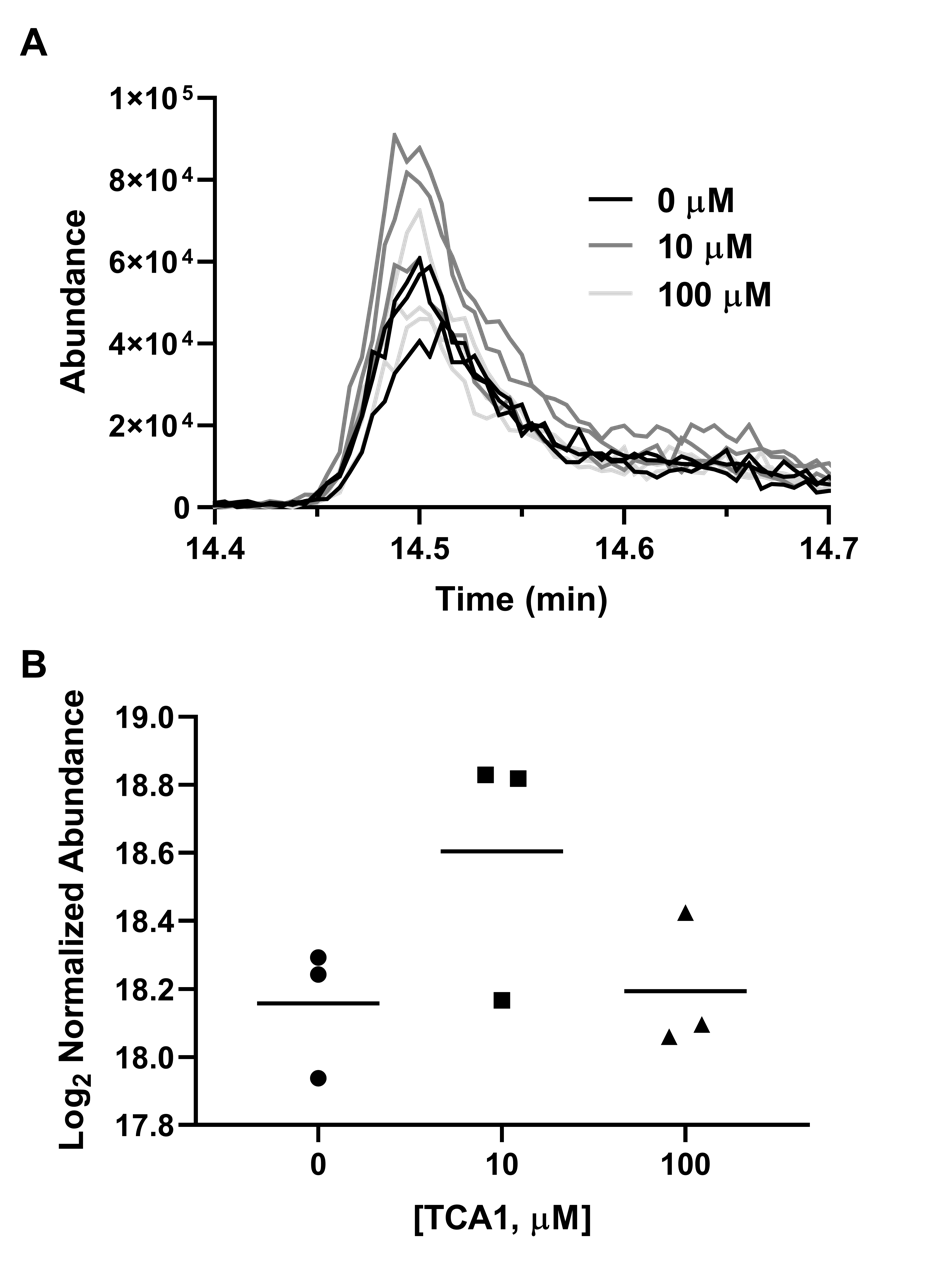


**Figure S12. Effects of TCA1 on Tyz synthesis using heterologously expressed enzymes.** (A) EICs corresponding to the 14.5 minute peak for C_12:0_-Tyz in reaction mixtures supplemented with 0 (black), 10 μM (dark grey) and 100 μM (light grey) TCA1. Reactions were performed in triplicate. (B) Log_2_ normalized abundance for integrated peak areas shown in panel A. Horizontal lines indicate the mean from reactions performed in triplicate, indicated by black symbols. A one-way ANOVA indicated there was no statistically significant difference between treatment groups (*p* = 0.16).

**Figure S13. MS/MS Fragmentation of the C_12:0_-Tyz isolated from *M. tuberculosis*.** The scan and fragmentation voltages of 10, 20 and 40 V are for the compound at ~14.6 min (Fig. 4A). Fragmentation is in agreement with data collected from C_12:0_-Tyz produced using *R. jostii* RHA1 extracts.

**
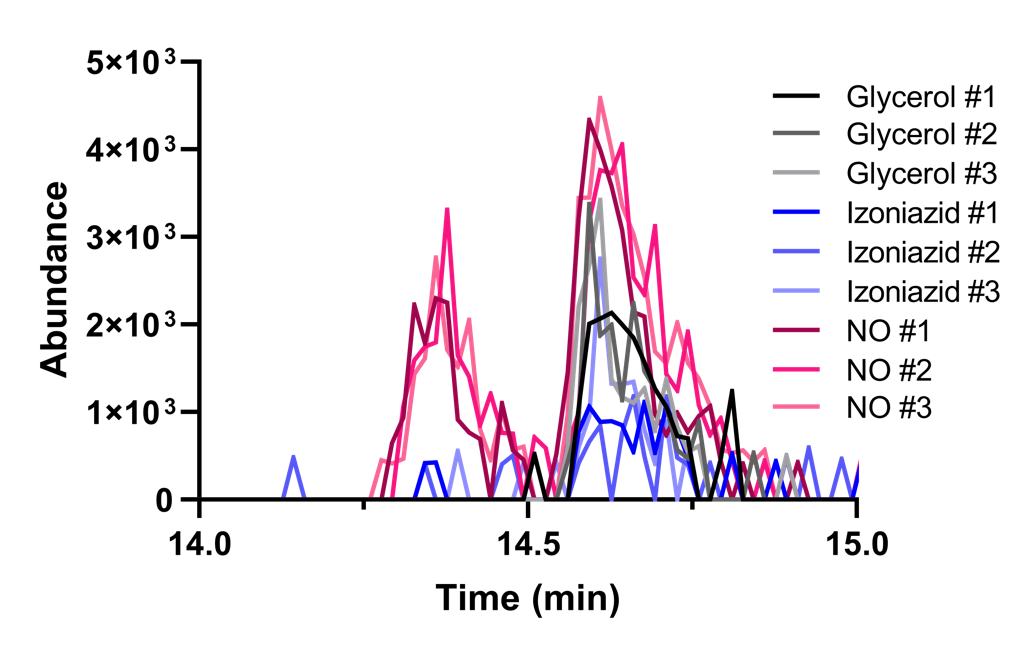
**

**Figure S14. The effects of NO and isoniazid stress on C_12:0_-Tyz production in Mtb.** EICs for the C_12:0_-Tyz in extracts from cells treated with no stressor (grey scale), NO stress (pink scale) or isoniazid stress (blue scale) in triplicate are shown. Final cell optical densities for the treatment groups were 2.6, 2.3 and 1.3 for glycerol, NO and isoniazid, respectively. Due to their detection around the limit of detection, accurate integration for statistical comparison was not possible.

**
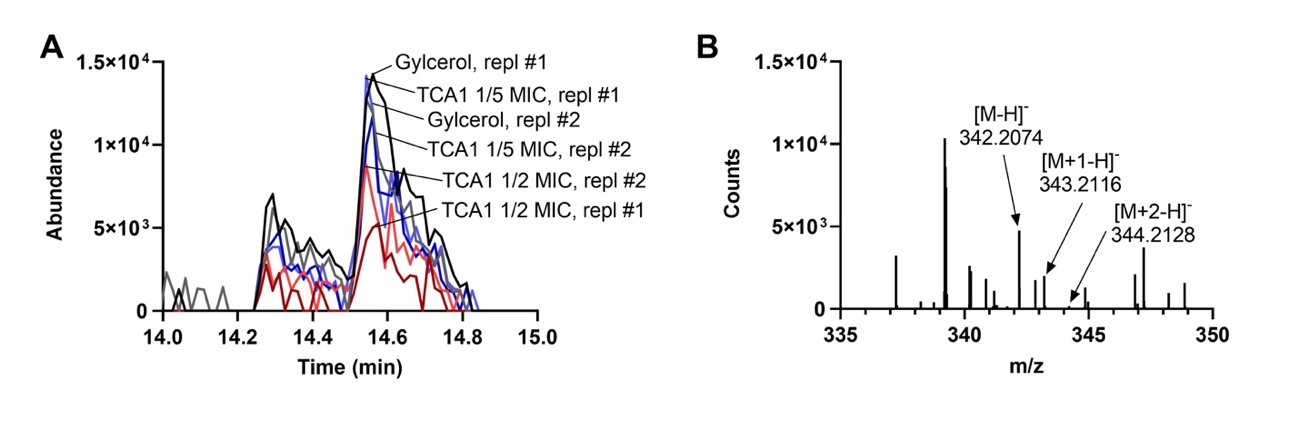
**

**Figure S15. Effects of TCA1 on production of the C_12:0_-Tyz in Mtb**. (A) EICs for the C_12:0_-Tyz detected in extracts from *M. tuberculosis* H37Rv strain mc^2^ 6206 treated with TCA1 at 0.2× (38 ng/ml) and 0.5× (95 ng/ml) its MIC value of 0.19 μg/ml (16). Two biological replicates are shown. (B) Representative mass spectra for the integrated peak at ~14.6 min in Panel A. Comparison of integrated peak areas for the duplicate biological samples did not reveal a statistically significant difference (one-way ANOVA, *p* = 0.075).

**Tables**

**Table S1. Major subclusters of FDOs identified in the SSN analysis.**

| Subcluster # | # of sequences | Mtb representative(s) |
| --- | --- | --- |
| 1 | 1998 | Rv2032 (Acg), Rv3127 |
| 2 | 1633 | -- |
| 3 | 1471 | -- |
| 4 | 1445 | Rv1355c |
| 5 | 1364 | Rv3131 |
| 6 | 784 | -- |
| 7 | 771 | -- |
| 8 | 623 | -- |
| 9 | 362 | -- |
| 23 | 83 | Rv2337c (TyzC) |

**Table S2. Bacterial strains and plasmids used in this study.**

|  | Description | Source |
| --- | --- | --- |
| **Strains** |  |  |
| *E. coli* DH5α | DNA propagation | -- |
| *E. cloni* | Protein production | Lucigen |
| *M. tuberculosis* mc^2^ 6206 | H37Rv, Δ*leuCD* Δ*panCD* | (32) |
| *R. jostii* RHA1 |  | (51) |
| *R. jostii*::*tyzA* | RHA1 with chromosomal insertion of *tyzA* | This study |
| **Plasmids** |  |  |
| pExpresso | Protein production, kanamycin^R^ | Lucigen |
| pExpresso-SUMO-TyzA | pExpresso harboring *tyzA* | This study |
| pTipQC2 | Protein production, chloramphenicol^R^ | (29) |
| pTip-H_6_-TyzA | pTipQC2 harboring *tyzA* | This study |
| pTip-TyzBC | pTipQC2 harboring *tyzBC* | This study |
| pRIME | Insertion vector, apramycin^R^ | (30) |
| pRIME-TyzA | pRIME harboring *tyzA* | This study |

**Table S3. Oligonucleotides used in this study.**

| **Name** | **Sequence** | **Restriction** |
| --- | --- | --- |
| pCDF-Rv2336-F | CTACCATGGTCAGATGTCTTGGATTCCTTCGCT | *NcoI* |
| pCDF-Rv2336-R | CGCTCGTATTAATTAATCAGATGTCTTGGATTCCTTCGCT | *PacI* |
| 2336-TEV-NcF | TCTACCTGCCATGGGCGAAAACTTGTATTTCCAGGGCATGGATGTCCCTCACGAG | *NcoI* |
| pTip-II-R | CACCGGCACCCGCAGCGA |  |
| pRIME_Rv2336-F | CTTTAAGAAGGAGATATACATATGGATGTCCCTCACGAG | *Nde*I |
| pRIME_Rv2336-R | CACGGGTGCCGGTGGGTCGACTAGTTCAGATGTCTTGGATTCCTTC | *Spe*I |
| pExpresso_2336_5p | CGCGAACAGATTGGAGGTATGGATGTCCCTCACGAGCAG | *--* |
| pExpresso_2336_3p | GTGGCGGCCGCTCTATTAGATGTCTTGGATTCCTTCGCTAAG | *--* |
| Rv2338_NdeI-F | CGATCATATGCGAGCCGGTGCGGATGA | *NdeI* |
| Rv2337_XhoI_R | CGTACTCGAGTTACGCGAAGCGGGATTCACTATCCG | *XhoI* |
